# Pretraining alpha rhythm enhancement by neurofeedback facilitates short-term perceptual learning and improves visual acuity by facilitated consolidation

**DOI:** 10.3389/fnrgo.2024.1399578

**Published:** 2024-06-04

**Authors:** Ming Chang, Shuntaro Suzuki, Takahiro Kurose, Takuya Ibaraki

**Affiliations:** ^1^Vie, Inc., Kamakura, Japan; ^2^NTT Data Institute of Management Consulting, Inc., Tokyo, Japan; ^3^ROHTO Pharmaceutical Co., Ltd., Osaka, Japan

**Keywords:** neurofeedback, alpha rhythm, perceptual learning, visual acuity, Gabor patch

## Abstract

**Introduction:**

Learning through perceptual training using the Gabor patch (GP) has attracted attention as a new vision restoration technique for myopia and age-related deterioration of visual acuity (VA). However, the task itself is monotonous and painful and requires numerous training sessions and some time before being effective, which has been a challenge for its widespread application. One effective means of facilitating perceptual learning is the empowerment of EEG alpha rhythm in the sensory cortex before neurofeedback (NF) training; however, there is a lack of evidence for VA.

**Methods:**

We investigated whether four 30-min sessions of GP training, conducted over 2 weeks with/without EEG NF to increase alpha power (NF and control group, respectively), can improve vision in myopic subjects. Contrast sensitivity (CS) and VA were measured before and after each GP training.

**Results:**

The NF group showed an improvement in CS at the fourth training session, not observed in the control group. In addition, VA improved only in the NF group at the third and fourth training sessions, this appears as a consolidation effect (maintenance of the previous training effect). Participants who produced stronger alpha power during the third training session showed greater VA recovery during the fourth training session.

**Discussion:**

These results indicate that enhanced pretraining alpha empowerment strengthens the subsequent consolidation of perceptual learning and that even a short period of GP training can have a positive effect on VA recovery. This simple protocol may facilitate use of a training method to easily recover vision.

## 1 Introduction

Vision is an essential sensory modality and an important element of daily life. Myopia, hyperopia, and age-related vision loss are common problems, resulting in widespread use of visual aids such as spectacles and contact lenses. Poor vision can interfere with daily activities such as reading, driving, and sports and has a significant impact on quality of life. Thus, methods for vision restoration have long received particular attention.

Traditional methods of vision restoration are dominated by invasive approaches such as surgery, laser *in situ* keratomileusis (LASIK), and refractive surgery. Although effective, these methods have disadvantages, such as the risks involved in surgery and their high cost. In addition, options are currently limited for patients for whom surgery is not applicable or who do not wish to do it.

Recently, an approach called perceptual learning has been suggested for vision restoration. Perceptual learning is the process of collecting input through sensory organs and processing it to improve sensory accuracy and perception. This approach is based on the plasticity of the visual system and involves repetition of specific tasks to improve visual function. This may allow for sustained improvements in vision, maintained with continued adequate training. Further, perceptual learning is noninvasive and has the advantage of avoiding the risks and costs of surgery. Furthermore, since it is individually adaptable, training programs can be designed to meet the patient's needs.

Recent vision research indicates that visual abilities such as contrast sensitivity (CS) and visual acuity (VA) can be trained by perceptual learning. CS refers to the visual ability to distinguish objects or images with different levels of brightness, which is crucial for visual tasks in daily life, such as reading under low light conditions or driving at night. Through perceptual learning, individuals can improve their ability to detect lower contrast stimuli. This enhancement in visual sensitivity allows for better detection of finer visual details. VA refers to the ability to see objects clearly at a specified distance; further, perceptual learning in general may be effective for treating amblyopia (Polat et al., 2004; Zhou et al., 2006). The neural basis of perceptual learning lies in the visual cortex, while enhancing brain noise elimination and optimizing information use may enhance visual organ capabilities (Levi and Li, 2009; Sagi, 2011; Lev et al., 2014). However, the severity of the original amblyopia and task training duration may be more involved in improved task performance after training than the task itself (Li and Levi, 2004; Li et al., 2005).

Among all perceptual learning tools, the Gabor patch (GP) has been widely used to enhance specific visual functions in visual tasks such as the VA by more efficiently processing retinal images blurred by presbyopia (Polat et al., 2004, 2012; Polat, 2008; Sterkin et al., 2018). Further, target recognition and detection training using two stimuli results in improved VA (Polat et al., 2004; Polat, 2008), contrast detection (Polat, 2009; Polat et al., 2009), contrast discrimination (Polat et al., 2012), and reading speed (Polat et al., 2012). Similarly, GP use has been shown to improve myopia maintaining this effect after 2 months (Durrie and McMinn, 2007; Tan and Fong, 2008; Camilleri et al., 2014b) and to improve CS, sports skills, and reading speed (Deveau and Seitz, 2014). Many studies have demonstrated that training with GP often concurrently enhances CS and VA, and the improvement in CS is closely associated with overall visual enhancement (Zhou et al., 2006; Tan and Fong, 2008; Levi and Li, 2009). In an experiment to measure differences in fMRI brain activity with two different GP tasks using the same visual stimuli, decoder construction after a GP experiment showed that decoders learned in the Gabor patch can function in complex natural images containing a variety of visual information (Tsushima et al., 2020). These suggest that the CS and VA improvement effect of the GP may be generalizable to daily visual perception. These findings suggest new prospects for the development of vision restoration technology using perceptual learning. Nonetheless, the perceptual learning task itself is monotonous and painful and requires a large number of training sessions and a relatively long time before being effective, which is an issue for interested users.

Neurofeedback (NF) is an innovative method leveraging the plasticity of the human brain to enhance cognitive ability. It involves monitoring the electrical activity of the brain via EEG and providing real-time feedback to individuals, enabling them to consciously influence their neural patterns (Sitaram et al., 2017). This technique has shown promising results in enhancing attention, memory, and other cognitive functions by targeting specific brain oscillations such as the alpha and beta frequencies. For instance, alpha NF training has been linked to improvements in memory and attention tasks, suggesting a direct correlation between increased alpha oscillations and enhanced cognitive performance (Nawaz et al., 2022; Li et al., 2023; Kimura et al., 2024).

Furthermore, brain oscillations also play a significant role in perceptual learning. Brain oscillations at specific frequencies in the alpha band are associated with learning and consolidation (Freyer et al., 2013; Muller-Gass et al., 2017). In addition, occipital transcranial alternating current stimulation (tACS) at 10 Hz, but not at 20 or 40 Hz, increased both learning rate and performance improvement during an orientation discrimination task (He et al., 2022). Moreover, a previous study has suggested that increasing the strength of alpha waves in the sensory cortex via neurofeedback (NF) before training for tactile perception can effectively facilitate perceptual learning (Brickwedde et al., 2019). However, the effect of alpha NF on perceptual tasks, especially regarding improvements in visual perception, is not yet fully established. No research has investigated whether NF could help restore vision.

Therefore, we conducted an experiment to verify whether enhancing the alpha power in the visual cortex with NF before visual training can improve the efficiency of visual training and restore vision in myopic participants throughout a short training period. All participants were enrolled in behavioral GB training, being randomly assigned to either the NF or control group. Based on previous research, we hypothesize that NF can boost and accelerate the effects of perceptual learning in myopic adults when combined with a short perceptual training regime. To investigate the effects of NF on visual learning, VA and CS were assessed for each participant before and after training.

## 2 Materials and methods

### 2.1 Participants

Twenty participants (age 20–49 years old, mean age: 27.35) with myopia were recruited for this study. The participants' binocular VA ranged from 0.125 to 0.9. They were randomly assigned to the NF (12 participants including eight males and four females; mean age: 26.17; mean VA: 0.51) or control (8 participants including 3 males and 5 females; mean age: 29.13; mean VA: 0.56) group. Informed consent was obtained from all participants prior to the study. All experimental procedures were approved by the Shiba Palace Clinic Ethics Review Committee. In addition, all procedures were in accordance with the Helsinki Declaration of 1964 and its later amendments.

### 2.2 Procedure

Before the experiment, each participant's individual alpha peak frequency (IAPF) was detected to tailor the NF training to their specific neural activity.

**Figure 1:**
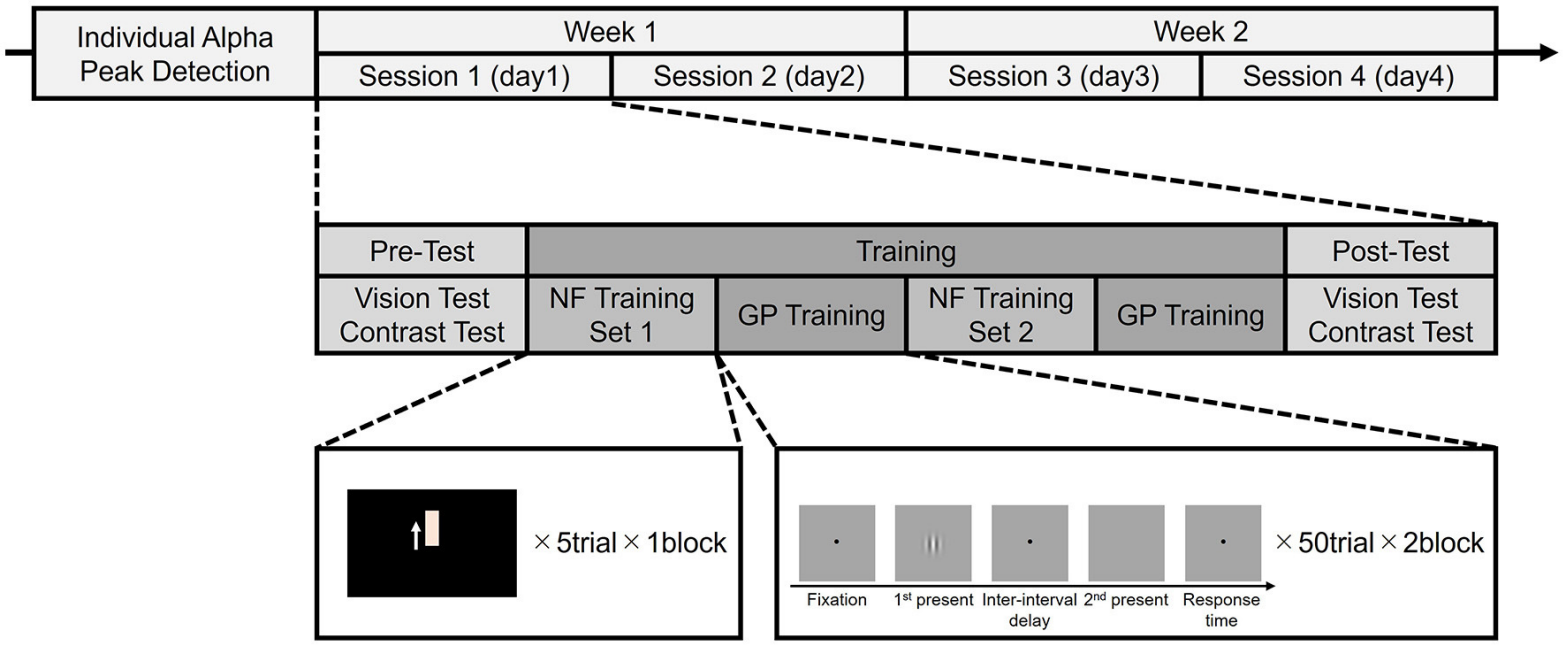
Flowchart of the experimental design and procedure.

The participants completed four sessions over 2 weeks, two sessions per week (Figure 1). Each session consisted of a pre-test, a training phase consisting of two sets of NF training and GP visual training, and a post-test. Furthermore, each session was held on a separate day to ensure recovery and prevent fatigue, allowing for consolidation of possible learning effects.

### 2.3 Pre and post-test

Participants underwent a pre-test including a VA test and a CS test to establish baseline measures. VA was determined using Landolt rings with orientations of 0°, 45°, 90°, 135, 180°, 225°, or 315°. The participants responded to the direction of the Landolt ring on the screen at a distance of 5 m. Methods and VA indices conformed to standard Japanese protocols. Initially, a stimulus with a decimal VA of 0.1 = 10 visual angle breaks in a Landolt ring is presented and the direction judged. After a correct answer, a ring one level smaller is presented; in case of an incorrect answer, a ring of the same size is presented again. After an incorrect response, if the participant's correct answer is still more than 50% of the total number of answers answered so far, a ring one step smaller was presented. Conversely, if the number of correct answers is <50%, a ring one step larger was presented, and the decimal VA was calculated based on the smallest visual angle at which participants could get >50% of correct answers.

CS was measured using a GP with a spatial frequency of 3° and 6°, respectively. GP was presented with an orientation of 0°, 45°, 90°, or 135°. As with the vs. test, participants responded to the direction of the GPs on the screen at a distance of 5 m. Participants underwent VA and CS tests again in the post-test phase to measure any effects from training.

### 2.4 NF training

NF training was conducted in one block of five trials to increase the alpha power before each GP vision training. Each trial consisted of a 30 s baseline measurement and 60 s NF training.

The baseline was determined using the absolute value of the Hilbert transform. Baselines were measured for 30 s, and moving averages were calculated every 0.5 s. Data > 3 standard deviations (SD) of the baseline were removed and the mean and SD of the baseline calculated. After baseline determination, the visual feedback displayed on a computer screen was a bar colored with a MATLAB copper color map. The feedback was administered for 60 s and updated every 100 ms. The bar expanded and contracted over a range of ±2 SD from the baseline, depending on the alpha power. The baseline was represented by a bar without length, a strong alpha power above baseline is indicated by a bright orange bar pointing upward, and weak alpha power below baseline is indicated by a dark orange bar pointing downward. Participants were asked to extend the bar on the screen as far up as possible. Data > ±5 SD from the baseline were removed; in such a case, the bar would disappear.

Participants in the control group did not undergo NF training; instead, they watched videos for a time equivalent to the NF training time.

### 2.5 GP training

Before GP training, participants were asked to adjust the GP contrast to the lowest contrast that could detect GP at a distance of 150 cm from the screen. Participants' adjusted contrast was used as the initial contrast for GP presentation at the beginning of GP training.

**Figure 2:**
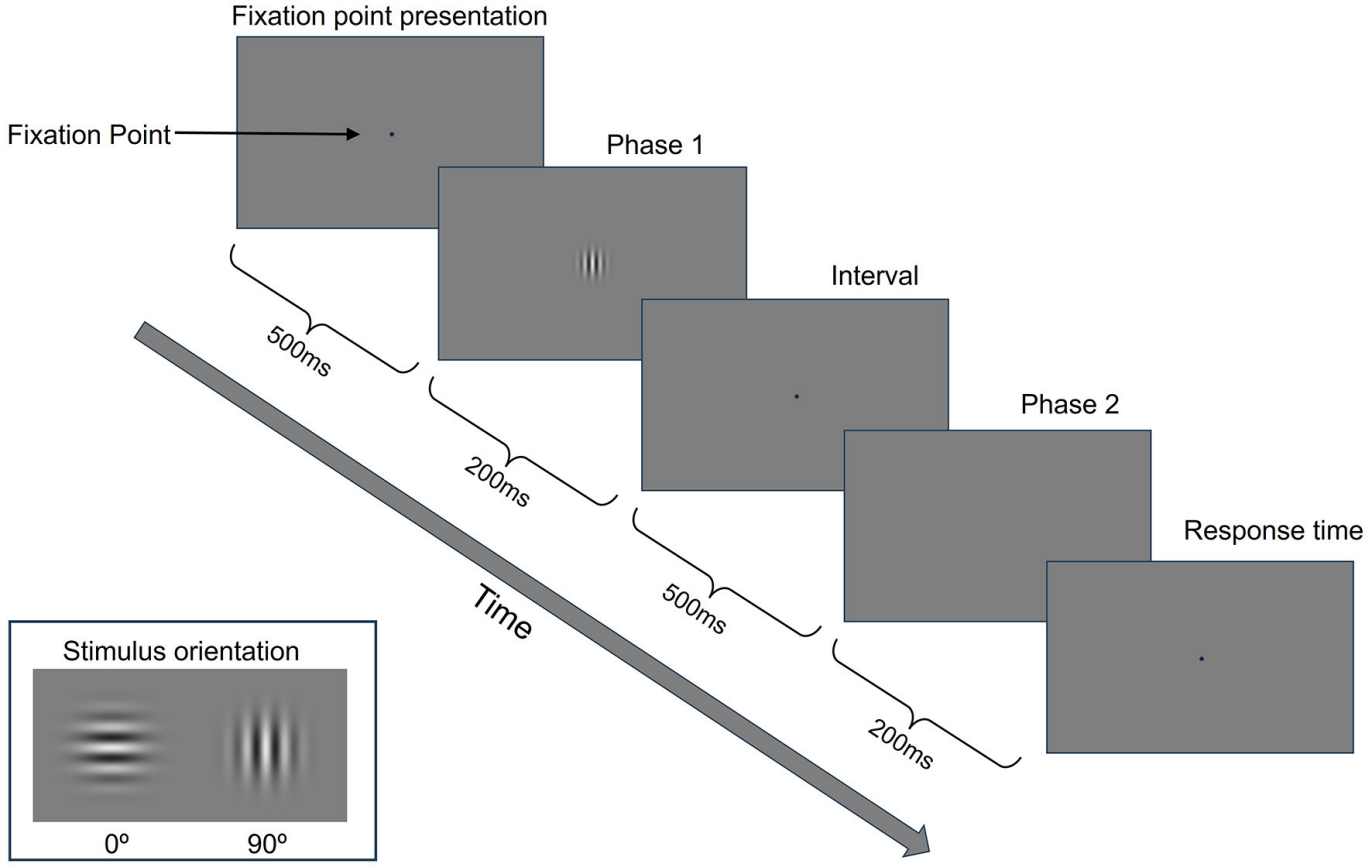
GP training paradigm. Participants must decide in which presentation phase (the first or second) the central Gabor target has been presented.

For GP training, we used a two-interval forced choice (2IFC) task to measure contrast detection thresholds for a single target (isolated stimuli) (Benhaim-Sitbon et al., 2023). As shown in Figure 1, GP training consisted of two blocks, each with 50 trials. Each trial comprised a fixation point presentation (500 ms), stimulus presentation phase 1 (200 ms), interval (500 ms), stimulus presentation phase 2 (200 ms), and response time (Figure 2). The stimulus was presented randomly in either phase 1 or 2. Participants were asked to choose the phase presenting the stimulus from the two phases at a distance of 150 cm from the screen. After getting ready, the participant presses any button on the numeric keypad to start a training block. The participant had to determine which of the two stimulus presentation phases contains the stimulus by pressing a numeric keypad button (num 1 for the first one, num 2 for the second one). There was no time limit for responses; the trial lasted until the participant responded. During stimulus presentation phases 1 and 2, an auditory tone was presented as a cue indicating when a stimulus might appear. After the participant responded, a green fixation point was displayed as feedback for the correct answer. For incorrect answers, a red fixation point and a beep sound were presented simultaneously. A 2:1 staircase procedure was used, i.e., the contrast level of the target increased 25% after each mistake and decreased by 25% after two consecutive correct responses. This staircase converged at a level of 70.7% correct (Levitt, 1971).

The stimulus orientation used in GP training was 0° in the first week and 90° in the second week (Figure 2). Considering effectiveness in enhancing contrast sensitivity and overall visual acuity, the spatial frequency of the stimulus used in GP training was 3 cycles per degree (cpd) in the first session and 6 cpd in the second session of each week (Tan and Fong, 2008; Deveau and Seitz, 2014).

### 2.6 Electroencephalographic recordings

Electroencephalogram (EEG) was recorded during NF training using a modified In-Ear EEG (VIE ZONE, VIE, Inc.). We recorded from an earphone-type electrode placed in the left ear canal and a solid gel electrode placed at Oz according to the international 10–20 system. The ground and reference electrodes were placed on the back of the left and right sides of the neck, respectively. The EEG data sampling rate was 600 Hz. Ultimately, the EEG ultimately was re-referenced by subtracting the signal from the left ear electrode from the signal from the Oz electrode.

For identifying IAPF, participants were required to maintain closed and open eyes for 20 s each, repeating this twice. The IAPF was considered as the alpha peak within the 8–13 Hz range. In NF training, the bandpass filter was set to IAPF – 1 Hz to IAPF + 1 Hz for each participant.

### 2.7 Statistical analysis

For the CS data of each GP frequency recorded in the first and last measurement, we normalized individual CS threshold in the last measurement after the training by the corresponding threshold in the first measurement obtained before the training; therefore, we can calculate the normalized improvement as:


$$\begin{array}{l} \text{Normalized Improvement}=\frac{\text{Post Threshold – Pre Threshold}}{\text{Pre Threshold}} \end{array}$$

The normalized improvement in CS in both groups was compared using an unpaired *t*-test. Similarly, we also calculated the normalized improvement in VA, and the normalized improvement in VA in both groups was compared using an unpaired *t-*test.

Moreover, to evaluate the impact of training on VA improvement, we similarly normalized individual VA which measured at the beginning and end of each training day by the corresponding initial VA in the first measurement obtained before the training. A one-factor repeated measures multivariate ANOVA was used to test whether the VA at each measurement point changed significantly from baseline. If significant effects were identified, a *post-hoc* test for multiple comparisons by Bonferroni was conducted within each repeated-measure ANOVA. The threshold for significance was set at *p* < 0.05.

To evaluate the effect of training aimed at increasing alpha power by NF, interactions were evaluated as for VA and power increase (single-tailed one-sample *t*-test with z-score 0 as reference) for the standardized power in each NF set (2 NF sets per day) based on the mean and variance of alpha power before NF.

In addition, to examine the effect of alpha power on the cross-day consolidation effect on VA, a single regression model was constructed to explain the initial VA of the next training day (difference from the previous post-training VA) using the mean alpha power of the previous NF session as a predictor (for all subjects in both groups).

## 3 Results

We investigated changes in CS at each GP frequency after training. Using CS data gathered during the pre-test and post-test phases, we compared normalized improvement in CS and VA in the NF and control groups, respectively.

**Figure 3:**
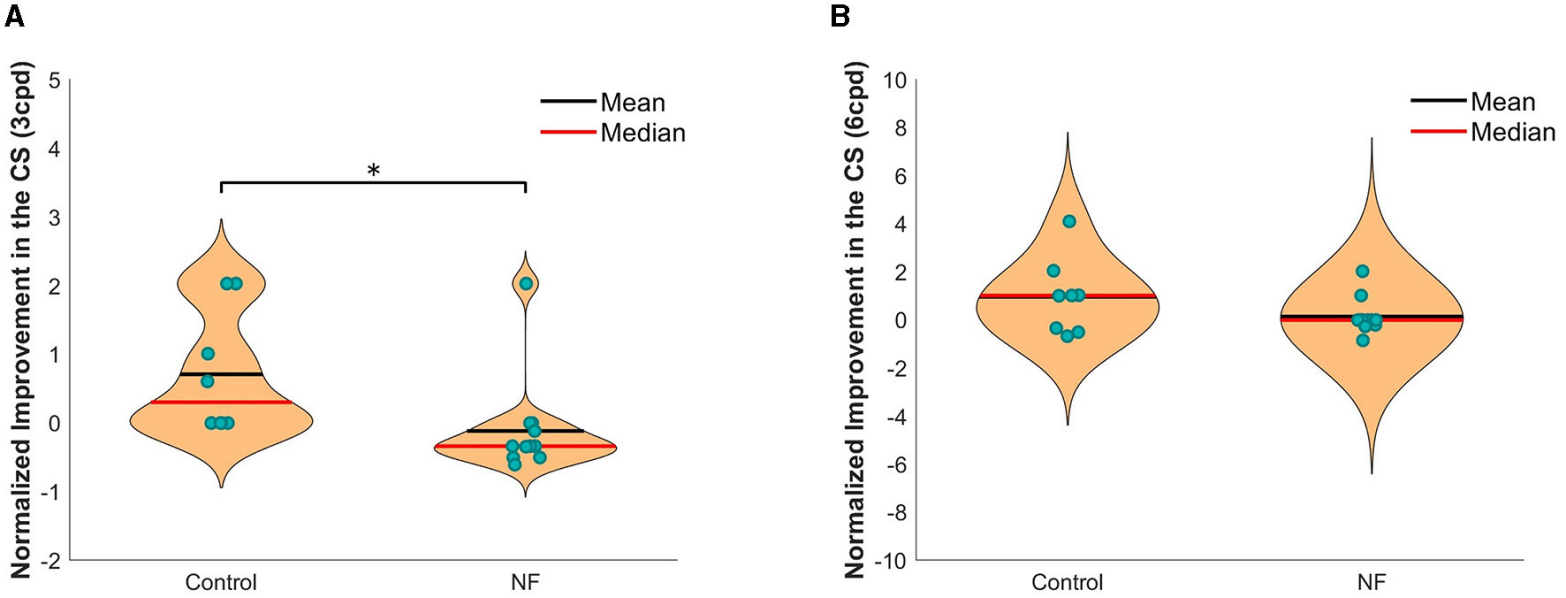
Normalized improvement in CS at 3 cpd **(A)** and 6 cpd **(B)** in both groups. *p < 0.05.

For the normalized improvement in CS, the unpaired *t*-test showed significant group difference at 3 cpd CS (Figure 3). The improvement for 3 cpd CS in the NF group was significantly greater than in the control group [*t*_(18)_ = −2.31, *p* < 0.05]. However, no significant group difference at 6 cpd CS was observed [*t*_(18)_ = −1.58, n.s]. In addition, to verify the CS improvement, we compared the original CS threshold from the first and last measurements of the two groups respectively (Supplementary Figure S1). For 3 cpd CS, a paired *t*-test showed significant improvement in the NF group [*t*_(11)_ = −2.43, *p* < 0.05], while no significant improvement in the control group (t_(11)_ = −2.13, n.s). However, no significant improvement was observed in either group compared with the first measurement in the 6 cpd CS.

**Figure 4:**
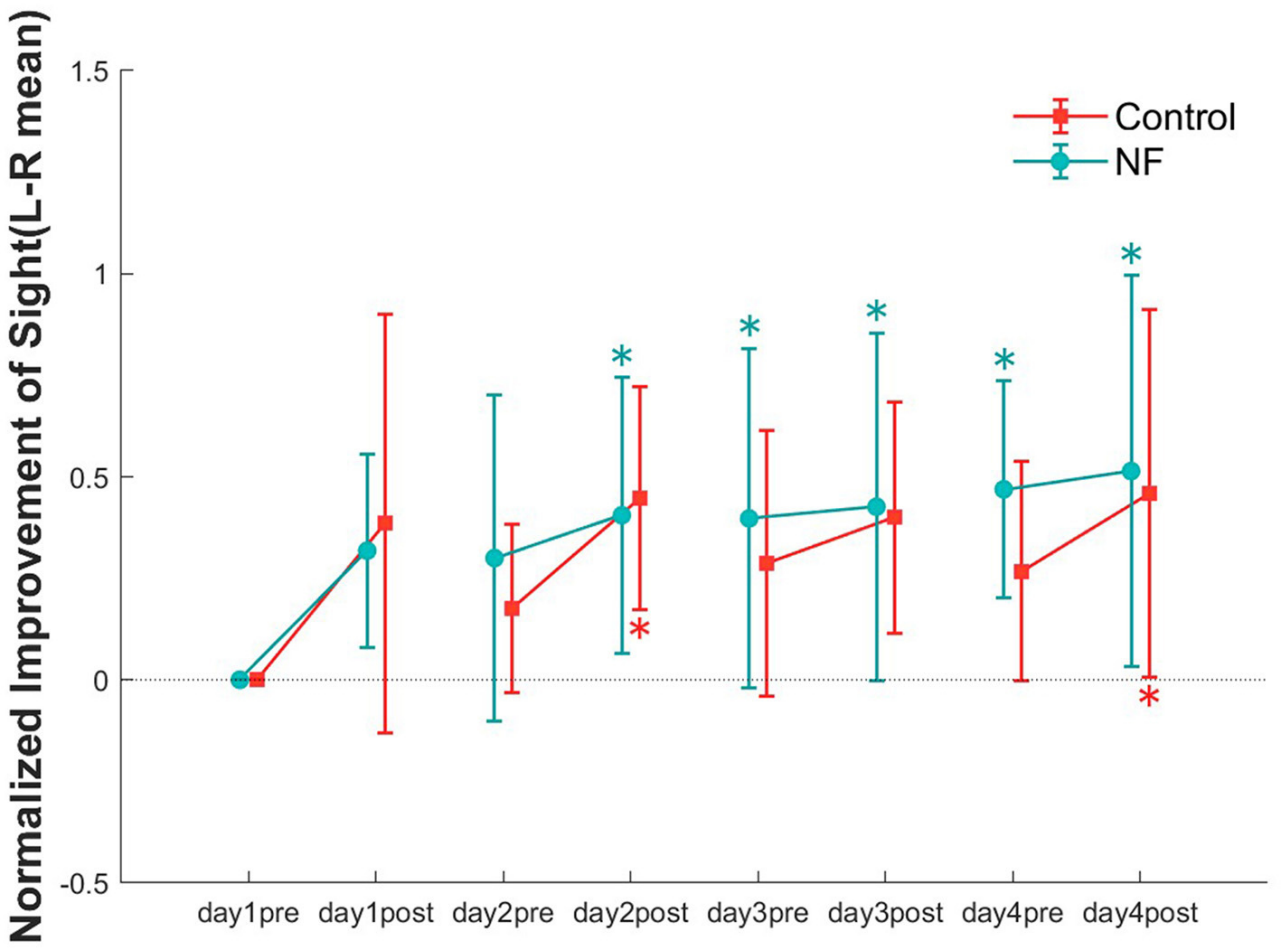
Normalized improvement in VA at all data points (pre and posttest of 4 days) in both groups. Error bars represent standard error of the mean (SEM). *p < 0.05.

To examine the effect of training sessions on VA, we compared the VA of each data point in each group (Figure 4). The repeated measures ANOVA showed significant improvement in VA in both groups with increasing training time [NF: *F*_(7, 77)_ = 4.26, *p* < 0.01; control: *F*_(7, 77)_ = 3.03, *p* < 0.05]. Although the unpaired *t*-test showed no significant difference for normalized improvement between the two groups [*t*_(18)_ = 0.25, n.s], the VA of the NF group was higher to that of the control group at every pretest. Regarding the main effect of the measurement point, the results of multiple comparisons indicated that a significant improvement in VA within the NF group at each measurement from the posttest conducted on the day 2 through to the last measurement (MSe= 0.0716, *p* < 0.05, alpha' = 0.0018). In the control group, significant improvement in VA was only observed in the post-tests on the day 2 and day 4 (MSe = 0.0644, *p* < 0.05, alpha'= 0.0018).

**Figure 5:**
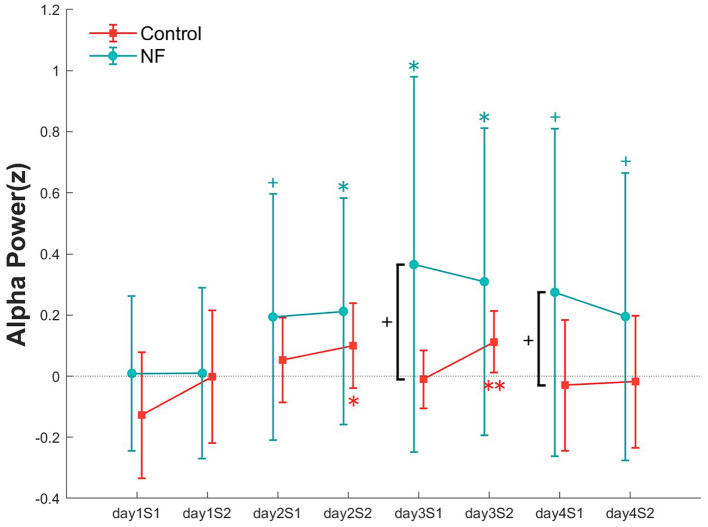
Alpha power at all NF sets over 4 days in both groups. Error bars represent SEM. ^+^*p* < 0.1, *p < 0.05, and **p < 0.01.

To examine the effect of NF on alpha power, we compared the alpha power in each NF training set for each group (Figure 5). For the alpha power, a repeated measures ANOVA showed no significant interaction [*F*_(7, 119)_ = 1.2, *p* > 0.05]. However, in the NF group, compared with the alpha power of the first set on day 1, all sessions from day 2 onward showed a significant or a marginally significant increase in alpha power. Similar to the VA results, the alpha power of the NF group recorded in each NF training session was higher to that of the control group; a marginally significant difference emerged in the first NF training sets on day 3 [*t*_(18)_ = 1.7, *p* < 0.1] and 4 [t_(18)_ = 1.52, *p* < 0.1].

**Figure 6:**
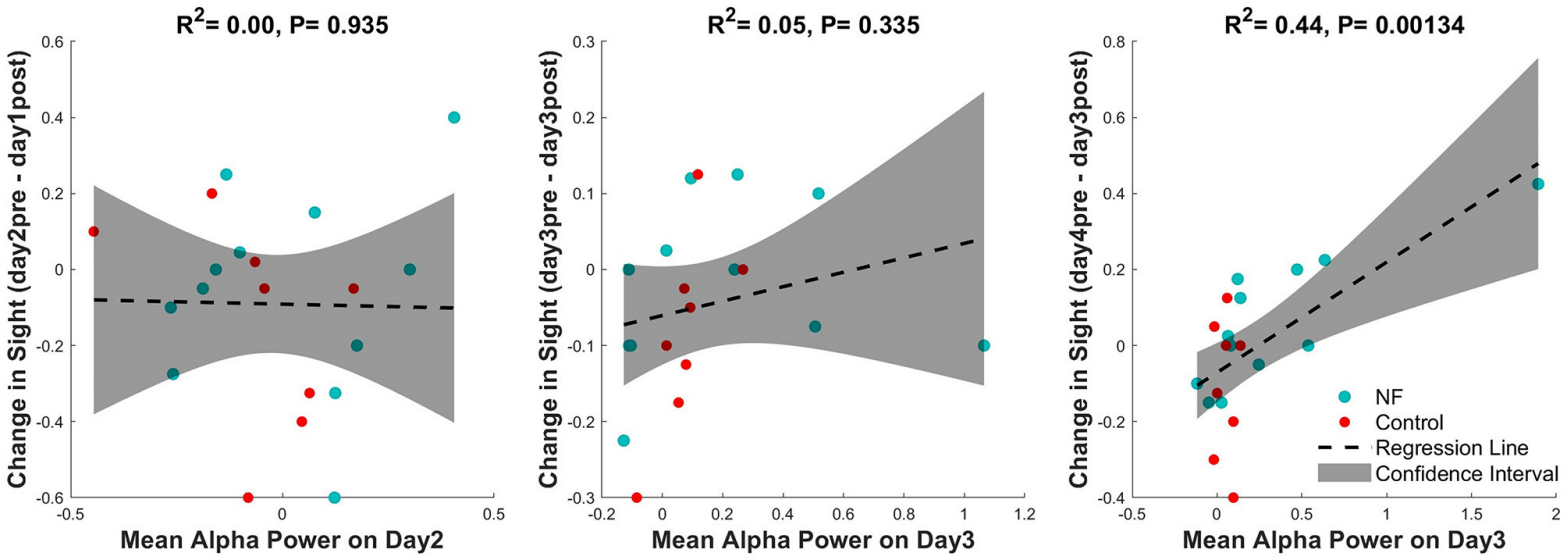
Correlation between alpha power in the previous session and the VA change from the posttest in the previous session to the pretest in the current session.

In the VA analysis, the NF group showed a significant improvement in vision in the pretest on days 3 and 4 compared with baseline. To investigate the relationship between this effect and alpha power, the alpha power from the previous session and the VA change from the posttest of the previous day to the pretest of the current day were analyzed for all participants. No significant correlation was observed on days 2 and 3, but on day 4, the alpha power from the previous session (day 3) predicted the change in VA (β = 0.29, *p* < 0.01; Figure 6).

## 4 Discussion

In this study, we introduced a novel approach to vision restoration, demonstrating that pretraining alpha rhythm enhancement via NF can significantly improve perceptual learning in myopic individuals. Participants were divided into NF and control groups, which differed as to whether GP training was preceded by NF training for enhancing alpha power. We observed a significant difference in 3cpd CS improvement between groups. The improvement for 3 cpd CS in the NF group was significantly greater than in the control group. Compared before training, the results of 3 cpd CS after training showed significant improvement in NF group, while no significant improvement in control group. However, the improvement and the group difference were not observed in 6cpd CS. In addition, although no significant differences for VA improvement were observed between the groups, only the NF group showed improvement in VA in the pretest on days 3 and 4. The correlation analysis showed that the alpha power observed during NF training on day 3 was significantly associated with vision recovery in the pretest on day 4.

The present study, focused on NF enhancement of alpha rhythm to improve VA and perceptual learning in myopic individuals, is supported by previous research. For instance, multisensory perceptual learning and sensory substitution can significantly impact neural plasticity, as evidenced by work with sensory substitution devices for the blind, where visual information is transformed into other sensory modalities such as sound or touch (Proulx et al., 2014). Furthermore, our study aligns with findings on the flexibility of the electrophysiological properties of sensory neurons in the adult cortex. Prior research has demonstrated that these properties can change in response to sensory input alterations, thereby affecting perceptual performance (Zohary et al., 1994). This aligns with our observation of improved VA and perceptual learning through NF-induced alpha rhythm enhancement, suggesting a direct link between targeted neural modulation and enhanced sensory performance.

Our research on NF-induced alpha rhythm enhancement in improving visual processing aligns with the growing body of neuroscience research, which indicates that brain wave patterns, particularly alpha rhythms, are closely linked to cognitive and perceptual functions (Herrmann et al., 2013; Sigala et al., 2014). A previous study indicated that pre-stimulus alpha oscillations play a significant role in visual perception through their interaction with gamma-band oscillations (Zazio et al., 2020). Notably, aligning learning tasks with an individual's natural brainwave rhythms was reported to significantly boost learning efficiency (Michael et al., 2023). By syncing participants' brain activity to their individual alpha wave frequencies, participants could learn cognitive tasks at least three times faster than without. This aligns with our findings that modulating alpha rhythms through NF improved visual learning. Moreover, our research extends existing knowledge by demonstrating the therapeutic potential of manipulating alpha oscillations. While previous studies focused on the natural oscillatory role of alpha rhythms in attention and perception (Snyder and Foxe, 2010; Brickwedde et al., 2019; Peylo et al., 2021), our work showcases the practical application of these rhythms in enhancing sensory functions, particularly in vision.

In our research, the control group showed significant improvement in VA only at two measurement points, all of which were found in the post measurement immediately after the training sessions, suggesting that the effects of GP training without NF were only temporary and did not persist to the next training day. Conversely, the NF group exhibited significant improvement in every measurement after the training on the day 2, including the pretests of the last two training days. This result suggests that enhancement of alpha power by NF has a consolidation effect on training outcomes, enabling the maintenance of improvements until the next training day. Moreover, we also uncovered that the power of alpha rhythms on day 3 showed a significant correlation with VA in the pretest at day 4. This suggests that the alpha state can predict subsequent perceptual performance. A previous study clarified the influence of brain states on individual differences in somatosensory learning. Based on EEG recordings before and during learning, it became clear that prelearning parietal alpha oscillations and stimulus-induced contralateral central alpha changes during learning can predict learning outcomes (Freyer et al., 2013). This is consistent with our results. Furthermore, a previous study found that alpha waves increase in the frontal and posterior lobe after completing a sequence learning task. These posttask changes may represent traces of motor learning and correlate with use-dependent plasticity (Moisello et al., 2013). Accordingly, it is possible that the increase in alpha power observed in our study helped participants retain traces of perceptual learning, to maintain the learning effects of the previous session until the next session; that is, the increase in alpha power had a consolidation effect on perceptual learning. However, we did not observe a significant association between alpha power and increased VA after the first two learning sessions, indicating that ≥3 learning sessions are required to achieve this consolidation effect.

The study findings offer promising implications for non-invasive myopia treatment. Although tACS are effective in regulating oscillatory activity in the human brain (He et al., 2022), the large extracranial electrical current applied in tACS is short-circuited by the skin, making it challenging to model and predict the exact intracranial current densities and electric fields (Herrmann et al., 2013). Despite the weak current tACS intensity, it is nonetheless an external electrical stimulation. NF presents an accessible, cost-effective alternative that leverages the body's neural plasticity, potentially benefiting a wider range of individuals, including those unsuitable for surgery. This approach aligns with modern trends in personalized medicine, emphasizing patient-friendly, tailored treatments. Furthermore, the use of wearable EEG technology, demonstrated in our study, offers a noninvasive, user-friendly approach to various neurological and sensory challenges in the clinic.

Compared with previous studies of visual training (Table 1), our visual training required fewer sessions and training time (Supplementary Figure S3), which may be due to the use of NF to enhance alpha power during training. However, our study has some limitations, such as the small sample size and short-term training, common challenges in neuroscience research. In future research, longer term studies with larger, more diverse participants, including different age groups and varying degrees of myopia, would be beneficial for understanding the long-term effects of NF-induced alpha rhythm enhancement in VA and perceptual learning. Nevertheless, the present results suggest that enhancement of alpha power by NF plays a role in maintaining training effects, which allows us to anticipate potential long-term effects. Furthermore, our findings provide innovative insights and approaches to vision science, raising the possibility of approaches to sensory enhancement and rehabilitation.

**Table 1:** Overview of visual training studies using GB.

**References**	**Participants (Visual impairment; Number of Participants; Age)**	**Training (Training period; Number of sessions; duration / 1 session)**	**Flanker**	**Outcome (Improvement VA within subject: pre vs post)**
Sterkin et al. (2018)	Myopia Initial VA: 0.00 0.22 LogMAR *N =* 32 Age: mean 47.8, SD± 3	Unknown (41 ± 12.7 sessions, (mean ± SD) At least 3 sessions a week) 12 min/1 session	Both	Improvement VA: 0.13 LogMAR 2-way ANOVA Main effect of training *F*_(1, 31)_ = 16.9, *p <* 0.001 / *t*-test before and after training *p =* 0.005
Camilleri et al. (2014b)	Myopia/Astigmatism Initial VA: −0.75 −2 D/less than −0.5D *N =* 10 Age: 22–27, mean 24.22	8 weeks (24 sessions) 45 min/1 session	NA	Improvement VA: 0.16 LogMAR 2-way ANOVA Main effect of training *F*_(1, 7)_ = 7.95, *p <* 0.05 / *t*-test before and after training *p =* 0.057
Moret et al. (2018)	Amblyopia Initial VA: More than 0.1 LogMAR *N =* 20 Age: 27–58, mean 44	2 weeks (8 sessions, Four consecutive days in a week × 2) 45 min/1 session	Y	Improvement VA: 0.193 LogMAR 2-way ANOVA Main effect of training *F*_(1, 18)_ = 19.088, *p =* 0.0001 / *t-*test before and after training *p =* 0.048
Polat (2009)	Presbyopia Initial VA: 0.385 LogMAR *N* = 13 Age: mean 50, SD± 1.1	10 weeks (20 sessions, 2 sessions a week) 30 min/1 session	Y	Improvement VA: 0.19 LogMAR (an average of 54%) Pre and posttraining *t*-test *p =* 0.035
Camilleri et al. (2014a)	Myopia/Astigmatism Initial VA: −0.75 −2 D/less than −0.5D *N =* 16 Age: 19–27, mean 24.12	2 months (8 sessions, Four consecutive days in a week × 2 and 6 weeks with different cpd for each subject) 45 min/1 session	NA	Improvement VA: 0.15 LogMAR Friedman's ANOVA before and after training χ^2^ = 10.57 *p <* 0.005
Casco et al. (2014)	Myopia Initial VA: 0.75 1.75D *N =* 7 Age: 20–25	6 weeks (12 sessions, 2 sessions a week) 30 min/1 session	Y	Improvement VA: 1.3 LogMAR 2-way ANOVA Main effect of training *F*_(1, 6)_ = 12.34, *p =* 0.013 / *t*-test before and after training p(2λ) = 0.001
Zhou et al. (2006))	Myopia Initial VA: 2.4 23.8 LogMAR *N =* 23 Age: 14–27, mean 19.3	Unknown (9-19 sessions, Average 12.7 sessions) 45 min/1 session	NA	Improvement VA: 4.6 dB / 0.58 LogMAR *t*_(6)_ = 4.38, *p <* 0.01; SE, 1.0 dB; range, 1.9–10.0 dB; median, 3.8 dB / r^2^ = 0.93, *p <* 0.01
Yalcin and Balci (2014)	Amblyopia Initial VA: Average 0.42 LogMAR *N =* 99 Age: 9–50, mean 20.3	4 months (45 sessions, 3 sessions a week) 30 min/1 session	Y	Improvement VA: 2.6 LogMAR Z =-3.24, p=0.001
Polat et al. (2004)	Amblyopia Initial VA: 0.41 ± 0.14 LogMAR *N =* 44 Age: mean 35, SD± 13	2 - 4 weeks [45 ± 15 sessions (mean ± SD)] 30 min/1 session	Y	Improvement VA: 0.18 LogMAR SE ± 0.1
Lunghi et al. (2019)	Amblyopia Initial VA: 0.1 0.78 LogMAR *N =* 10 Age: 27–40, mean 32.9	4 weeks (6 sessions) 2 h/1 session	NA	Improvement VA: 0.15 ± 0.02 LogMAR *t*_(9)_ = 7.7, *P <* 0.001
Durrie and McMinn (2007)	Myopia/ Presbyopia Initial VA: 0.20 0.60 LogMAR *N =* 11 Age: 19–39, mean 31.4	2 −3 months (30 sessions, 2 - 3 sessions a week) 30 min/1 session	Y	Improvement VA: 2.2 LogMAR Unknown
Current study	Myopia Initial VA: 0.05~0.9 LogMAR *N =* 11 Age: 20–49, mean 26.89	2 weeks (4 sessions, 2 sessions a week) 30 min/1 session	NA	Improvement VA: 0.16 LogMAR *t*_(11)_ = 4.05, *p <* 0.01

NA, Not available; Y, Yes.

## Data availability statement

The original contributions presented in the study are included in the article/Supplementary material, further inquiries can be directed to the corresponding author.

## Ethics statement

The studies involving humans were approved by the Shiba Palace Clinic Ethics Review Committee. The studies were conducted in accordance with the local legislation and institutional requirements. The participants provided their written informed consent to participate in this study. Written informed consent was obtained from the individual(s) for the publication of any potentially identifiable images or data included in this article.

## Author contributions

MC: Data curation, Formal analysis, Investigation, Methodology, Validation, Visualization, Writing – original draft, Writing – review & editing. SS: Investigation, Resources, Software, Validation, Writing – original draft. TK: Funding acquisition, Writing – review & editing. TI: Conceptualization, Data curation, Formal Analysis, Funding acquisition, Investigation, Methodology, Project administration, Resources, Software, Supervision, Validation, Visualization, Writing – original draft, Writing – review & editing.
