# Supplementary material for: Pretraining alpha rhythm enhancement by neurofeedback facilitates short-term perceptual learning and improves visual acuity by facilitated consolidation
Source: Front Neuroergon. 2024 Jun 4;5:1399578. doi: 10.3389/fnrgo.2024.1399578 (PMC11184131; doi:10.3389/fnrgo.2024.1399578)
Supplement: Supplementary file 1 [file Data_Sheet_1.PDF]

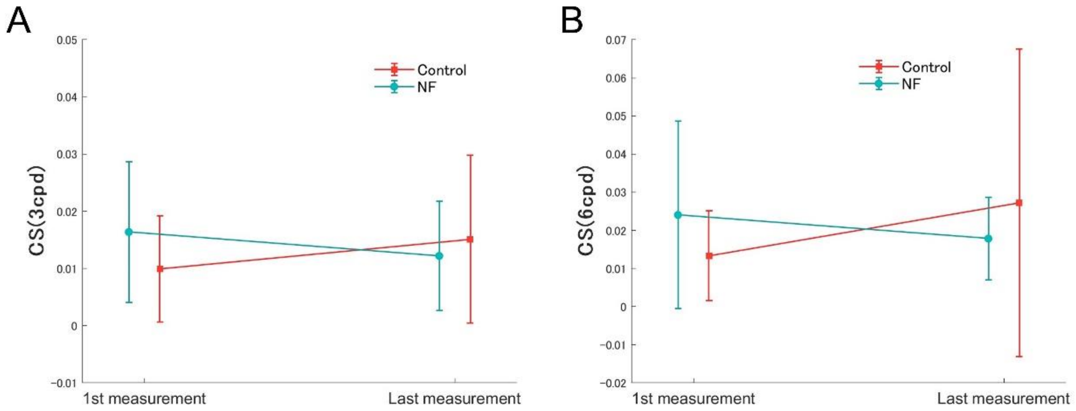

Fig S1. CS at 3 (A) and 6 cpd (B) for first measurement (baseline) and last measurement in both groups

Unpaired t-tests confirmed that the differences in baseline values of CS between the two groups were not statistically significant (CS(3cpd): t (18) =1.25, p = 0.23; CS(6cpd): t (18) =1.14, p = 0.27).

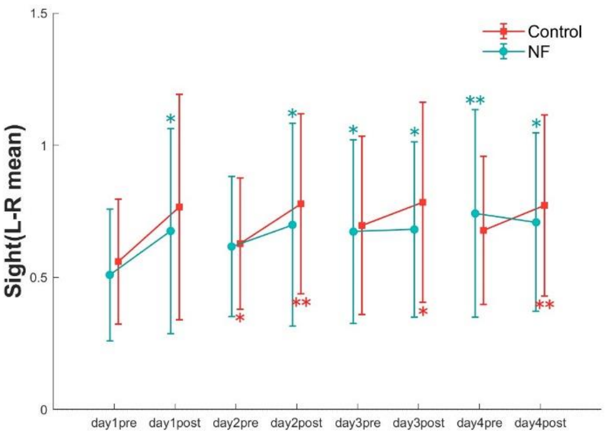

Fig S2. VA at all measurement points (pre and posttest of the 4) in both groups

Unpaired t-tests confirmed that the differences in baseline values of VA between the two groups were not statistically significant (t (18) = -0.45, p = 0.66).

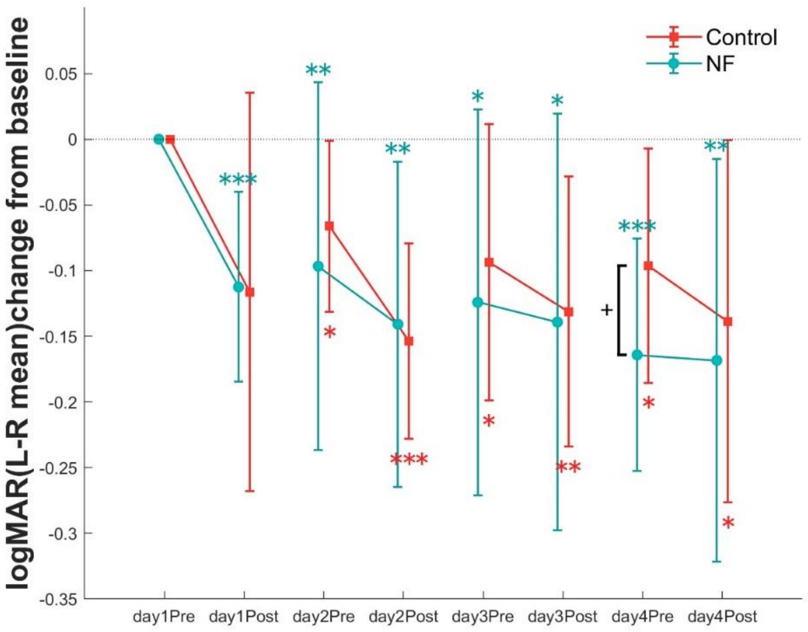

Fig S3. Change in logMAR at all data points (pre- and posttest of 4 days) from the first measurement in both groups
